# Special Issue “Raman Spectroscopy and Machine Learning in Human Disease”

**DOI:** 10.3390/ijms262311529

**Published:** 2025-11-28

**Authors:** Ivan Bratchenko

**Affiliations:** Scientific and Educational Center “Fundamental and Applied Photonics. Nanophotonics”, Immanuel Kant Baltic Federal University, Kaliningrad 236041, Russia; iabratchenko@gmail.com

Nowadays, modern optical techniques offer a variety of approaches for the analysis of tissues and biofluids [1,2,3]. Raman spectroscopy stands out among optical methods as it provides fast, precise, and rapid analysis without complex sample preparation [4]. These advantages have broadened our perspectives on human disease research. In recent decades, researchers have analyzed different diseases based on the evaluation of Raman spectra. Such studies have covered almost the entire field of medicine, including optical biopsy for the detection of cancers [5], heart and vessel diseases [6], infectious diseases [7], and many other diseases [8,9,10]. The complex spectral data acquired in the experiments requires proper evaluation and analysis. In this regard, machine learning techniques, including artificial intelligence and neural network applications, are widely used in the analysis of optical biopsy data [11].

The articles in this Special Issue demonstrate a diverse range of applications of Raman spectroscopy and machine learning techniques. Two review papers [4,12] have emphasized the use of Raman spectroscopy in thyroid nodule evaluation and medical applications for diagnostic tasks. As a result, the authors concluded that Raman spectroscopy, combined with machine learning, “have powerful application potential,” but, at the same time, they also highlighted “the remaining challenges and limitations preventing their translation into clinical settings.”

Since every disease changes the metabolic profile of the patient, changes in the chemical components of the affected tissues or biofluids may be tested with Raman spectroscopy. Raman spectroscopy highlights changes in the “spectral biomarker” associated with the development of the disease (in this case, registered spectrum serves as a biomarker). Six research articles have focused on different applications of Raman spectroscopy, each investigating the changes in the “spectral biomarkers” associated with different diseases.

The application of Raman microspectroscopy has provided an opportunity to classify myocardial tissue into muscle, necrotic, granulated, and fibrotic tissue types using collagen as a molecular biomarker. Principal component analysis (PCA) and support vector machines (SVMs) have been applied for dimensionality reduction and classification, with nonlinear models specifically addressing the nonlinearity of collagen formation during fibrogenesis [13]. As a result, the authors proposed a promising method for the monitoring of cardiac tissue state.

The use of dried samples for cerebrospinal fluid Raman spectra registration paved the way for the estimation of “spectral biomarkers” for Alzheimer’s disease [14]. Projection on latent structures with discriminant analysis (PLS-DA) was used for the dimensionality reduction and classification of the collected spectra. The permutation tests provided valuable statistical information on the reliability and stability of the developed PLS-DA models. The authors demonstrated high efficiency in Alzheimer’s disease identification, reaching an impressive 0.99 ROC AUC.

The utilization of surface-enhanced Raman spectroscopy (SERS) provides an opportunity for ultra-sensitive detection, even of tiny concentrations of components [5]. This approach was utilized for the analysis of urine to detect renal cell carcinoma [15]. Application of the support vector machine led to the creation of a sensitive technique that was able to detect the presence of renal cell carcinomas with 100% accuracy.

The combination of conventional Raman spectroscopy with a one-dimensional convolutional neural network provided 89.9% accuracy and 91.4% precision in colorectal cancer detection [16]. To achieve this goal, the authors utilized PCA to facilitate discrimination between malignant and normal tissues and to highlight their biochemical differences using loading plots.

Confocal Raman microspectroscopy provided an opportunity for in vivo analysis of skin tissues to detect patients with atopic dermatitis [17]. The authors demonstrated decreased total intercellular lipid and carotenoid concentrations, as well as a shift towards decreased orthorhombic lateral lipid organization in lesional atopic skin. The important result of this study is that information contained in the “spectral landscape” may be utilized not only for the classification (or detection) of disease but also for the development/control of targeted therapies.

Analysis of circulating cell-free DNA (ccfDNA) with Raman spectroscopy demonstrated great potential for the monitoring and detection of diseases [18]. The authors established reference Raman spectra of ccfDNA samples from healthy males and females with different conditions, including cancer and diabetes, extracting information about their chemical composition. For example, comparative observations showed a distinct spectral pattern in ccfDNA from breast cancer patients taking neoadjuvant therapy. Raman spectroscopy thus proved once more to have significant potential in the determination of specific disease markers.

The studies highlighted above not only demonstrated the superior performance of the combination of Raman spectroscopy and machine learning techniques; they also paid close attention to the validation and verification of the obtained results. Validation plays an important role in the prevention of classification and regression model overestimation. Without validation, numerous studies dedicated to the optical biopsy of diseases would demonstrate overoptimistic results [19,20,21]. Thus, we believe that this Special Issue will not only provide new ideas for the creation of optical biopsy approaches but will also make more researchers aware of the necessity of rigorous spectral data analysis and proposed model validation.
